# Cost per case or total cost? The potential of prevention of hand injuries in young children – Retrospective and prospective studies

**DOI:** 10.1186/1471-2431-8-28

**Published:** 2008-07-07

**Authors:** Elinor M Ljungberg, Katarina Steen Carlsson, Lars B Dahlin

**Affiliations:** 1 Department of Hand Surgery, Malmö University Hospital, Malmö, Sweden; 2 Lund University Centre for Health Economics, LUCHE, Lund, Sweden; 3 Vardal Institute, Lund University, Lund, Sweden

## Abstract

**Background:**

Health-care costs for hand and forearm injuries in young children are poorly documented. We examined costs in 533 children injured years 1996–2003.

**Methods:**

Health-care costs and costs for lost productivity were retrospectively calculated in children from three catchment areas in Sweden. Seven case categories corresponding to alternative prevention strategies were constructed.

**Results:**

Over time, diminishing number of ward days reduced the health-care cost per case. Among children, the cost of lost productivity due to parental leave was 14 percent of total cost. Fingertip injuries had low median costs but high total costs due to their frequency. Complex injuries by machine or rifle had high costs per case, and despite a low number of cases, total cost was high. Type of injury, surgery and physiotherapy sessions were associated with variations in health-care cost. Low age and ethnic background had a significant effect on number of ward days.

**Conclusion:**

The costs per hand injury for children were lower compared to adults due to both lower health-care costs and to the fact that parents had comparatively short periods of absence from work. Frequent simple fingertip injuries and rare complex injuries induce high costs for society. Such costs should be related to costs for prevention of these injuries.

## Background

Hand and forearm injuries in young children range from simple wounds, burn injuries and fractures to nerve and tendon injuries. Even in children severe complex injuries may occur. The most frequent injury among children, caused by jamming indoors, is a fingertip and nail bed injury with or without fracture. Recent research reports a substantial increase in incidence of children admitted with hand and forearm injuries in Sweden 1987–2001 [1,2]. The reasons for the increase are unknown. Health-care utilization of hand and forearm injuries in children is poorly documented [3-9]. In adults, the costs for a hand injury vary with type of injury and are influenced by several factors including the length of sick leave [10-12]. From a policy point of view it is useful to relate the cost of health-care utilization to the cost for prevention, not the least since many of the injuries in children probably can be avoided [3,13,14]. The information should be presented to politicians and authorities in view of the reported observed increased incidence. Our idea is that if injuries are to be prevented, the injury mechanisms behind different common or severe diagnoses must be identified. With experience from our earlier studies [1,2,15], we have created seven case categories, typical for hand and forearm injuries in children. In this study we quantify how much these different cases cost in relation to each other. With this information, injury prevention may be more effective and directed against the important cases from a cost point of view.

## Methods

The information about health-care resource use for hand and forearm injuries in children was collected during two previous studies [2,15]. Between 1996 and 2000 [2] we retrospectively included all young children (0–6 years old) with an unintentional hand or forearm injury (N = 455) that were treated as outpatients or hospitalized at the Department of Hand Surgery, Malmö, Sweden. The children were identified from the diagnostic registers by the Swedish Version of the International Classification of Diseases, Ninth Revision (ICD-9) during 1996–1997 (800A-999X) and Tenth Revision during 1998–2003 (S00.0-T98.3). Children with fractures of the radius and ulna or more proximal bones were not included, as they were treated at the Department of Orthopaedic Surgery. Children with bites and infections, due to trauma, were included.

During one year (February 2002 to January 2003), all children (N = 96) referred to the Department of Hand Surgery, Malmö, Sweden, were invited to a prospective study where a questionnaire was completed by the parents at the hospital (response rate 78/96:81%) [15]. From the questionnaires we had information on if, and for how long, each of the parents was absent from paid work as well as their normal profession and occupation. The cost of lost productivity was calculated from the number of days the parents stayed at home to take care of their injured child using the human capital approach [16]. The projects were carried out in compliance with the Helsinki Declaration and after approval by the Ethical Committee in Lund, Sweden (LU 707-01, LU 451-03; verbal and written informed consent).

We also compare the cost of health-care to the cost of prevention using an illustrative example of fingertip injuries caused by jamming in doors and prevention by installing doors with safety arrangements. The calculations were based on the Malmö catchment area where we had a complete coverage of hand injuries.

The costs within the health care sector were calculated using the fees paid by a referring hospital for patients registered outside the region of Skåne and included overhead cost using year 2000 prices. The fees were EUR 868 per emergency ward day, EUR 414 per elective ward day, EUR 231 per emergency operative minute, EUR 12 per elective operative minute, EUR 154 per physician visit and EUR 62 per visit to a physiotherapist or occupational therapist (annual average exchange rate year 2000: 1 EUR = 8.4465 SEK).

Details of health-care costs were retrieved from the patient's notes. In addition to this, we collected information about national background, age at the time of injury, sex, date and place of injury, aetiology and diagnosis. Data were also divided by the catchment area, i.e. Malmö municipality (all types of injuries referred to our department), county Skåne excluding Malmö municipality (at time of data collection more severe injuries referred to our department) and southern part of Sweden excluding Skåne (more severe injuries referred to our department).

Based on the diagnoses and the mechanisms of injury, and with experience from our earlier studies, we constructed seven case categories that correspond closely to alternative prevention strategies. These were; A) burn injuries caused by hot objects, B) fingertip injuries caused by jamming in doors or other pinch objects, C) fractures, dislocations and sprains caused by falls or hits, D) tendon and nerve injuries and wounds caused by sharp objects, E) complex injuries (an injury to more than one of the anatomical components of the hand or total or subtotal amputation through the middle or proximal phalanges) caused by falls with sharp objects, F) complex injuries caused by machines and rifles and G) other injuries (which include burn injuries not caused by hot objects, fingertip injuries caused by sharp objects and falls or hits, complex injuries and fractures caused by pinch or crush objects, infections and bites). The study includes all 533 children from 1996–2000 and 2002–2003.

Initial analyses of data showed a linear association between health-care cost and length of stay, but with an increasing variation around the mean trend. Separate cost functions were therefore estimated for three subsamples, following the length of hospital stay: no ward days, 1–3 ward days and 4 or more ward days.

### Statistical analyses

Data is described using median with 25^th ^and 75^th ^percentiles for the total sample and by catchment area. Multiple regression analysis [17] was used to analyse what factors were associated with the variation in costs. Regression analyses are used to describe the statistical associations between a dependent and one or more independent variables. The estimated coefficient of a variable is then the partial effect of that variable on the dependent variable holding all other variables constant. The distribution of health-care costs was skewed to the left with a tail of high-cost observations and the cost equations were estimated using the logarithmic transformation on cost [17]. The estimated coefficients then show in percentage terms how much higher or lower health-care costs were associated with different characteristics, holding everything else constant, i.e. the marginal effects. For instance, from Table 2 column (1), children with no ward days who had utilised physiotherapy during the health-care episode cost on average 56 percent more than other children with no physiotherapy, all else equal. As both dependent variable cost and independent variable length of stay were logged in the cost equations, the coefficient was an elasticity. For instance, from Table 2, column (2) the coefficient of length of stay is 0.70, which means that when length of stay increased by 10 percent, the cost increased by 7 percent, all else equal. Below each regression table we have listed the characteristics of the reference person. A reference person is a "baseline person" or a statistical construction used to make comparisons, but the study population does not necessarily contain a child with the listed characteristics.

The negative binomial regression model was used in the analysis of factors associated with the variation in length of stay. The incidence rate ratios (IRR) from the estimation show the relative effect of a characteristic holding other factors constant. An IRR equal to one means the same relative incidence and IRR less (greater) than one means a relatively lower (higher) incidence.

All analyses started by including all the available variables that were hypothesised to have an influence on costs. The least significant variable was then excluded and the model rerun. This procedure continued until only variables significant at the 5% level remained (reduced model) [11,12].

## Results

### Annual median cost and length of stay

**Figure 1:**
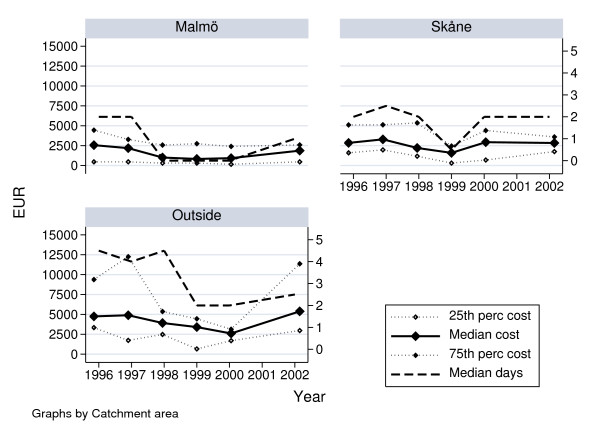
Annual median total health-care cost in EUR (left hand side scale) with 25^th ^and 75^th ^percentiles and number of ward days (right hand side scale) per patient 1996–2003.

A total of 533 children with an age up to 7 years were included in the study, consisting of 212 girls (40%) and 321 boys (60%). The distribution of health-care cost among the children by catchment area is shown in Table 1. The annual health-care cost for all injuries decreased during the study period from EUR 398,762 (1996) to EUR 247,540 (2000), and was EUR 192,624 for the 12 month period February 2002 – January 2003 by year 2000 prices of resource use. The number of patients during the years varied, i.e the highest number of treated patients were 2000 and the lowest 1996 and 2002–2003, with a median number of patients of 86 per year. The annual distribution of median cost per patient and length of hospital stay divided by Malmö municipality, Skåne (Malmö municipality excluded) and southern part of Sweden (excluding Skåne) is shown in Fig 1. Generally, the cost was higher and the length of stay longer among the patients from the area outside Skåne, compared to the patients from Malmö, during the entire period.

**Table 1:** The health-care cost per child, in EUR, of treating hand and forearm injuries in 533 young children at Department of Hand Surgery, University Hospital, Malmö, Sweden by catchment area.

	**Malmö **	**Skåne^1 ^**	**Southern Sweden^2 ^**	**Total**
	**(N = 323)**	**(N = 168)**	**(N = 42)**	**(N = 533)**
**Gender:**		*Number of children (%)*		

Boys	201 (62)	98 (58)	22 (52)	321 (60)
Girls	122 (38)	70 (42)	20 (48)	212 (40)
				
**Age:**		*Median age in months (25^th ^to 75^th ^percentile)*		

	45 (24–66)	38 (20–62)	40 (19–56)	42 (22–63)
				
**Cost data:**		*Median cost in EUR (25^th ^to 75^th ^percentile)*		

Length of stay	868 (0–1736)	2743 (0–2604)	2584 (1736–4341)	1282 (0–2150)
Surgery	188 (0–656)	563 (0–1061)	908 (352–1852)	351 (0–882)
Physician visit	308 (154–616)	462 (308–616)	308 (154–616)	308 (154–616)

Total health care cost	1280 (308–2814)	2743 (1049–4560)	3754 (2478–6486)	2102 (462–3606)

Data are presented as median with 25^th ^and 75^th ^percentile.

^1 ^Malmö excluded.

^2 ^Malmö and Skåne excluded

The main contributor to the total health-care cost was ward days followed by surgical sessions and physician visits. Only 12% of the children used physiotherapy (<1% of the costs; not included in Table 1). The median cost of surgical sessions was higher for patients from Skåne, and particularly for patients from southern Sweden. This was largely explained by the fact that only major hand injuries were referred to the Department of Hand Surgery in Malmö, while the less severe injuries were treated at the local hospital. The impact of catchment area from the regression analysis is reported below.

**Figure 2:**
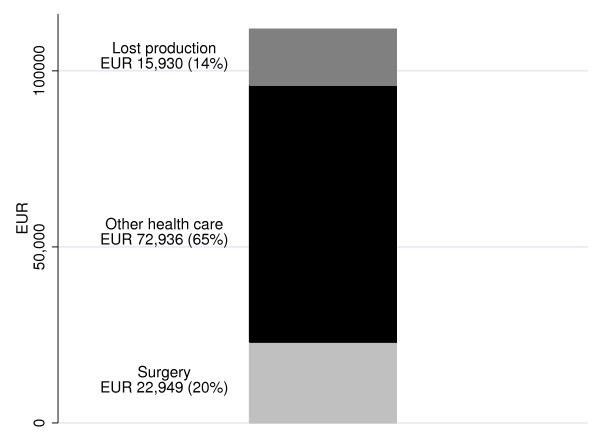
Total cost (EUR) for hand and forearm injuries in 51 children from the Malmö catchment area for 12 months 2002–2003 by sources of cost (surgery, other health-care cost, including ward days; lost productivity when parents were absent from paid work due to the child's injury).

In the subsample of children admitted in 2002–2003 and residents in Malmö (N = 51), we found that the cost of lost productivity was only 14 percent of the total cost, whereas the cost of surgical sessions amounted to 20 percent and other health-care costs to 65 percent (Fig 2).

### A. Burn injuries caused by contact with hot objects

**Figure 3:**
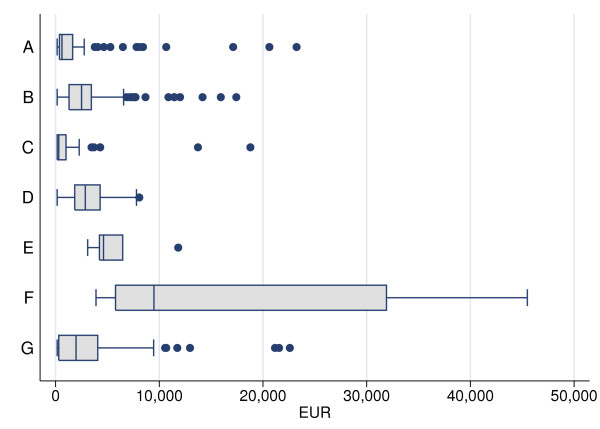
**Box plot of health-care cost (EUR) by type of case, sorted by median cost.** Seven case categories that closely corresponded to alternative prevention strategies were constructed: A) burn injuries caused by hot objects, B) fingertip injuries caused by jamming in doors or other pinch objects, C) fractures, dislocations and sprains caused by a fall or hit, D) tendon and nerve injuries and wounds caused by sharp objects, E) complex injuries (an injury to more than one of the anatomical components of the hand or total or subtotal amputation through the middle or proximal phalanges) caused by falls with sharp objects, F) complex injuries caused by machines and rifles, and G) other injuries.

**Figure 4:**
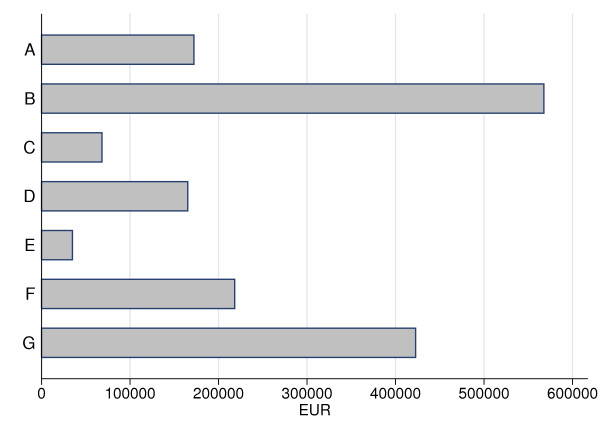
**Bar plot of health-care cost (EUR) by type of case, sorted by total costs for all patients.** Seven case categories that closely corresponded to alternative prevention strategies were constructed: A) burn injuries caused by hot objects, B) fingertip injuries caused by jamming in doors or other pinch objects, C) fractures, dislocations and sprains caused by a fall or hit, D) tendon and nerve injuries and wounds caused by sharp objects, E) complex injuries (an injury to more than one of the anatomical components of the hand or total or subtotal amputation through the middle or proximal phalanges) caused by falls with sharp objects, F) complex injuries caused by machines and rifles, and G) other injuries.

**Figure 5:**
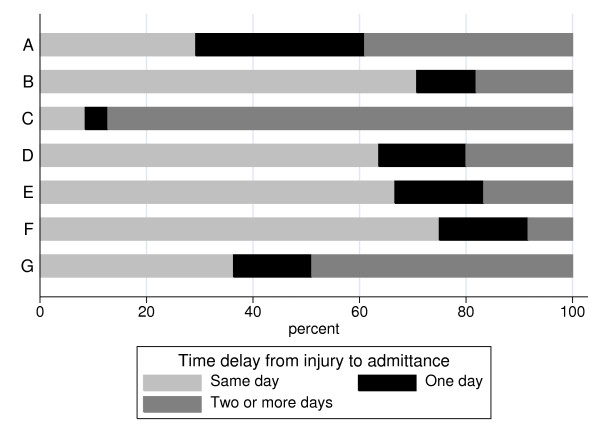
**Bar plot of distribution of time from injury to admittance by type of case sorted by percent admitted the same day, after one day and after two or more days.** Seven case categories that closely corresponded to alternative prevention strategies were constructed: A) burn injuries caused by hot objects, B) fingertip injuries caused by jamming in doors or other pinch objects, C) fractures, dislocations and sprains caused by a fall or hit, D) tendon and nerve injuries and wounds caused by sharp objects, E) complex injuries (an injury to more than one of the anatomical components of the hand or total or subtotal amputation through the middle or proximal phalanges) caused by falls with sharp objects, F) complex injuries caused by machines and rifles and G) other injuries.

The median cost per patient among burn injuries (N = 82) caused by contact with hot objects was low. A few children were admitted, needing multiple dressings and treatment with antibiotics, reflected by the number of outliers (Fig 3). The total health-care cost was therefore higher than for cases with fractures, dislocations and sprains caused by falls or hits (C) and complex injuries caused by falls with sharp objects (E); (Fig 4). The children were often admitted after more than one day (Fig 5).

### B. Fingertip injuries caused by jamming in doors or other pinch objects

The median cost for a fingertip injury (N = 188), caused by a door or any other pinch object, was in the middle compared to the other cases (Fig 3). In contrast, the total health-care cost for these cases was the highest, because of their frequency (Fig 4). More than 70% of these children were admitted to the department on the same day as the injury occurred (Fig 5). A patient with a fingertip injury, treated more than 4 ward days, had a low health-care cost compared to the reference case (Table 2).

**Table 2:** Ordinary least squares regression results of the logarithm of health-care costs on case categories, demographic and treatment characteristics.

Variable	(1)^a) ^No ward days Coefficient	(2)^b) ^1–3 ward days Coefficient	(3)^c) ^4+ ward days Coefficient
Fingertip injury caused by door or other pinch object			-0.10*
Fracture, sprain or dislocation caused by fall/hit	-0.56***		-0.16*
Tendon or nerve injury or wound caused by sharp objects	-0.75***	0.09*	
Complex injury caused by fall with a sharp object		0.32**	
Complex injury caused by machine or rifle		0.36*	0.28**
Other injuries	-0.39***		
Number of surgical sessions	1.02***	0.13**	0.04*
Number of physiotherapy sessions	0.56***	0.21***	0.18**
Logarithm of length of stay		0.70***	0.79***
Constant	5.99***	7.24***	7.29***

Number of individuals	213	218	102
Adjusted R^2^	0.35	0.69	0.89
F	23.83***	82.40***	179.89***
Akaike's information criterion	368.71	-87.08	-35.50

Separate regressions by length of stay: No ward days, 1–3 ward days and 4 or more ward days. Estimated coefficients denote percentage change in cost.

Note: Significance levels indicated by stars: * *p *< 0.05; ** *p *< 0.01 and *** *p *< 0.001.

Initial model controlled for age, gender, ethnicity, all case categories, physiotherapy, place of injury, catchment area, admitted on weekend, admitted later than the same day, surgery and length of stay. Insignificant variables were omitted from the final model.

^a) ^Reference person was a child with a burn injury or finger tip injury (categories A and B), no physiotherapy and no operation session.

^b) ^Reference person was a child from categories A, B, C or G, no physiotherapy, no operation session and zero ward days.

^c) ^Reference person was a child from categories A, D, E or G, no physiotherapy, no operation session and zero ward days.

### C. Fractures, dislocations or sprains caused by falls or hits

A fracture, dislocation or sprain (N = 47), caused by a fall or hit, had the lowest median cost (Fig 3). Thus, the total health-care cost for these cases was the second lowest of all categories, despite their high frequency (Fig 4). Almost 90% were admitted to the department two or more days after they were injured, since such injuries generally were not associated with open wounds (Fig 5). Outpatients and children treated more than 4 days were associated with low health-care costs, compared to the reference case (Table 2). The children were treated in about half the number of ward days as the reference case, probably since no operation was required (Table 3).

**Table 3:** Negative binomial regression of factors associated with the variation in number of ward days. All patients.^a)^.

Variable	IRR	95 % confidence interval
Fracture, sprain or dislocation caused by fall/hit	0.45**	0.27–0.75
Age	0.99***	0.99–1.00
Immigrant background	1.23*	1.00–1.51
Number of surgical sessions	3.46***	2.96–4.06
Admitted day after injury	0.77*	0.58–1.01
Admitted two or more days after injury	0.78*	0.63–0.98
Patient from Malmö city	0.58***	0.48–0.71

Alpha	0.53	0.41–0.67
Number of observations	533	
Pseudo R^2^	0.18	

Note: Significance levels indicated by stars: * *p *< 0.05; ** *p *< 0.01 and *** *p *< 0.001. IRR = incidence rate ratio.

^a) ^Reference person was a newborn child from categories A, B, D, E, F, G; from outside Malmö city and with Swedish background, admitted the same day as the injury and no operation session.

### D. Tendon and nerve injuries or wounds caused by contact with sharp objects

The median cost for a tendon/nerve injury or a wound caused by a sharp object (N = 55) was higher than for many other cases, but few cases were really expensive (Fig 3). The total health-care cost was about the same as for burn injuries caused by contact with hot objects (A), but not so high compared to complex injuries caused by machines or rifles (F) or to fingertip injuries (B; Fig. 4). The health-care cost was substantially lower for such a case not hospitalized (Table 2), including for instance a simple wound that did not need further treatment. Among admitted patients treated less than 4 days, the cost was high compared to the reference case, which may be reflected by the tendon and nerve injuries and not wound per se (Table 2). These cases were admitted early after the injury occurred (Fig 5), and were not associated with any significant change in length of stay (Table 3).

### E. Complex injuries caused by falls with sharp objects

The median cost of a complex injury caused by a fall with a sharp object (N = 6) was the second highest (Fig 3). These cases were few and, in contrast, the total health-care cost was the lowest when compared to all other cases (Fig 4). Two thirds were admitted the same day as they were injured (Fig 5). The cost analysis showed that the health-care cost was high compared to the reference case, if the patient was admitted less than 4 days (Table 2).

### F. Complex injuries caused by machines or rifles

A complex injury caused by a machine or a rifle (N = 12) entailed a high cost per case (Fig 3). Despite the low number of injuries, this group had the second highest total health-care cost of the defined categories [excluding the category Others (G)] (Fig 4). These children were admitted to the Department within one day after the injury (90%; Fig 5), often with an urgent transport by ambulance. In the cost analysis, the cost for such a case was substantially high compared to the reference case, among admitted patients (Table 2). These cases were not associated with any change in length of stay (Table 3).

### G. Other injuries

This category consisted of 143 patients, each with different injuries to the hand or forearm (see Methods). These cases were too few each to be separately included in the analyses. Generally, these heterogeneous cases had low median costs but with a few outliers with high costs. The health-care cost was low compared to the reference case, if the patient was not admitted (Table 2).

### Age, gender and national background

An increasing age was associated with less ward days (Table 3), but age had no significant effect on the health-care cost. We found no difference between girls and boys considering health-care costs or length of stay. Having an immigrant background was associated with more ward days (Table 3), but had no significant effect on health-care cost.

### Surgical sessions, physiotherapy, time before admittance and catchment area

Additional surgical and physiotherapy sessions were associated with higher health-care costs, mostly so for children treated as outpatients (Table 2). For example, compared to the reference person, having one additional surgical session was associated with a 102 percent higher cost [column (1)] and more ward days (Table 3).

### Cost of health-care and cost of prevention

In the light of impact on the use of health-care resources, we further explored the cost of prevention of fingertip injuries as an example. Based on communications with two manufacturers of doors, information was obtained on the additional cost of doors equipped with safety arrangements on the hinge side to prevent children from accidentally crushing their fingers. The additional cost ranged from EUR 85 to EUR 167 per door (prices Jan 2007). If all fingertip injuries could be avoided through installation of safer doors, treating 188 children at our department is equivalent to the additional cost of 3400 to 6680 doors with safety arrangements.

Continuing our example, we restrict the sample to Malmö municipality, where we were certain that all pinch injuries were transferred to our department. Assume that we wanted to avoid the 115 pinch-cases whose health-care costs were EUR 320 000 and using the less costly safety door at the addition cost per door of EUR 85, we would get 3765 doors for the same price. Malmö municipality had 133 000 households in 2003, and assuming that an average household has at least 5 doors, 665 000 doors would have to be replaced counting doors only in private homes. This calls for selective prevention strategies where safety doors are primarily installed in places where young children typically stay.

In our sample, house doors were by far the most common mechanism, but 13 of all children were injured in car doors. Hence, full prevention need to extend beyond houses. Alternative strategies could be closer surveillance of young children, temporary removal of doors indoors where children play, etc, or putting a towel on the top of the door thereby preventing the children to fully close the door [15]. Intervention programs by home visits can reduce the total costs of care [13].

In our data, 12 of the pinch-injuries in Malmö happened at the day care centre or school, while 46 occurred at home and 29 during leisure time. The mean number of children that went to day care centres in Malmö, between 1996 and 1999, was 10 870 [18], and the mean population in Malmö was 17 590 children (1–6 years old; ). Our estimate is that only a few children with hand injuries were injured at a day care centre, compared to the fact that 62% of all children went to day care centres during our study period. One explanation may be that safety doors are already installed at day care centres and schools in Sweden.

## Discussion

The main sources of health-care cost for 533 young children with hand and forearm injuries, treated at the Department of Hand Surgery, Malmö, Sweden, were ward days (59%) and surgery sessions (26%). In the subsample of 77 children, treated during the last year of the study (2002–2003), we had additional information on consequences of the injury measured by parents' days of absence from work due to the child's injury, a factor not previously considered [4,6,19]. The cost to society in terms of lost productivity, when parents stayed at home, accounted for 14% of the total cost and was about equal in magnitude to that of outpatient physician visits. Previous analyses on adults with tendon, nerve and other hand and forearm injuries from our department, showed long periods of sick-leave, implying that the cost of lost productivity accounted for 60–87 percent of total costs [11,12,20].

This may reflect at least two differences between children and adults. First, the fact children were able to attend the day care centre and adapt their play to the conditions of the injury. Hence, the injury had less consequence due to adaptable "usual activities". A few adults in the previous studies had a job where the hand injury caused less impairment, but in the typical case, workers were not able to perform their normal tasks at work and were thus granted sick leave. Secondly, eleven (14%) mothers and fathers reported that they were on parental leave at the time of the injury and thus no additional sick-leave was granted, as the parent was already at home taking care of the injured child and his/her siblings. As we measured lost market productivity, potential limitations in the family's activities were not included in the valuation. Only eight children (1.5%) were classified to have a major impairment with *potential *influence on future labour-market consequences, but we do not have further follow-up data on these children and hence we refrain further quantification in terms of costs or future lost quality of life. The analysis did not include quality of life issues, and will thus to some extent underestimate benefits of preventive strategies. In principle, the cost to the child in terms of the reduction in opportunities to play etc could also be included, but it was beyond the scope of this study to quantify the limitations in terms of type and quantity of activities of the child.

Our data covered a seven year long time period, and we found that length of stay has varied over time with a possible long-term downward trend (Fig 1) in spite of an increased incidence of hand and forearm injuries [1,2]. In particular, there was a dip in 1998–2000 for patients from the town of Malmö and in 1999 for patients from other parts of the region of Skåne. The decrease in length of stay for patients from other parts of southern Sweden dipped in 1999, but seemed to remain at the new level thereafter. Notably over the whole study, 42 patients were admitted from other county councils and the first year included 16 of these.

Fig 1 also illustrates the close relationship between length of stay and cost, which was consistent with our finding that ward days were the major source of cost for most patients (Table 2). We have used the price per ward day and other resource use from year 2000 paid by other county councils, which includes overhead costs. Over the period 1996–2003, the routines or criteria for admittance to or discharge from our department have not changed for health-care reasons. However, the organization of the health-care delivery changed when the Region Skåne was formed Jan 1 1999, merging the two county councils and one municipality formerly responsible for providing health-care. This coincided with the mentioned dip in length of stay for children with a hand or forearm injury, while the impact on median total health-care costs was smaller. Interestingly, from Fig 1 we also note that the patterns of change differ somewhat between the three catchment areas covered by the Department of Hand Surgery in Malmö. The median length of stay for children from Malmö and from the area outside Skåne decreased by two days implying an administrative decrease of length of stay, which did not seem to be true for children from Skåne (excluding Malmö). For all years but 1999, the distribution of length of stay and of cost per episode was not equal for patients from Malmö compared to the two other catchment areas by the Mann-Whitney test of equality of distribution. Patients from outside Skåne were not equal to patients from Skåne excluding Malmö in year 1996, while the lack of significance for years 1997 and onwards in this case was probably also related to the lack of statistical power as the number of patients from outside Skåne was below 10 these years. In the latter group, the number of actual nights in hospital may be overestimated for patients living in Malmö, who during the episode needed multiple re-dressings performed during anaesthesia, but otherwise were on leave from the hospital. Using a unit price per ward day the total health-care cost of these patients will be overestimated to some degree.

In a sensitivity analysis, we re-estimated the cost and length of stay equations, restricting the sample to only Malmö patients consisting of 61 percent of the total sample. We found, in essentials, the same factors to be associated with the variation in cost and length of stay. Neither did the magnitude of the coefficients change for central variables including the case categories.

It seems unlikely that changes in the administrative price per se during the observed period would have affected the incentives for referral and/or discharge. There have been no relative changes in the administrative price, only an increase by index. We cannot rule out, that constrained budgets of referral departments may have induced a reduced probability to refer children with less severe injuries, and rather treat them at home. Furthermore, the number of ward days for all patients, irrespective of age, has steadily decreased 1992 to 2006 by 1.0 day with no obvious dip. Hence the general trend seems to have affected also the number of ward days for young children and in some cases more so.

Our goal in the analysis has been to identify and quantify the cost to the health sector, and to society, of hand and forearm injuries in young children. Information on sources of cost and its magnitude is necessary for the selection of efficient prevention strategies, besides knowledge about the efficacy and other aspects of alternative interventions [3,4,21]. The cost of illness methodology used here is typically used in exploratory phases, where the aim is to develop and chose between different lines of development. For us this was also the motive behind defining case categories, rather than merely performing the analyses by diagnosis. As defined, the case categories are associated with the mechanism of injury, and thus possible preventive strategies.

In fingertip injuries caused by jamming in doors or other pinch objects (category B), the median was lower than tendon and nerve injuries and wounds, but a high number of treated patients implied that treatment of these fingertip injuries accounted for the single largest source of cost. The total health-care cost was EUR 568 000 for 188 patients. In comparison, the less common complex injuries caused by a machine or a rifle (category F), had substantial median cost per case but as there were only 12 patients over the entire period, the total health-care cost remained at EUR 218 000. This reflects the dilemma of prevention – cost per case or total costs [19].

Contact with sharp objects is the second most common (21%) injury mechanism in Swedish children (0–6 years) admitted for hand injuries [1]. In our cost analysis, patients with tendon/nerve injuries and wounds or complex injuries caused by sharp objects were associated with 9% and 32%, respectively, higher health-care costs for children treated 1–3 ward days. Such objects cause injury to important either single (tendon/nerve) or multiple (complex) anatomical structures. The health-care cost for complex injuries caused by machines or rifles was higher for children admitted 1–3 days (36% higher) and 4 or more days (28% higher). Among 10,000 Swedish children (0–6 years old) injured during 15 years, five percent of the injuries were caused by contact with machinery and 0.4% by contact with handgun/firearm discharge (severity unknown) [1].

Statistics from the National Board of Health and Welfare in Sweden (2003–2004) show that among children (0–17 years old) that applied for health-care at Emergency Departments or General Practitioner centrals, at hospitals, because of an injury, one fourth (23%) had injuries to the hand and wrist . Only injuries to the head were more common (25%). Separate information is not available for 0–6 year old children. The average incidence of hospitalized hand and forearm injuries among children 0–6 years was 40/100,000 persons/year during 1987–2001 and increased during this period [1]. The hand must be looked upon as a frequently recurrent part of the body in accidents among Swedish children. The cost point of view that we now have introduced is one way to bring the problem to general notice.

## Conclusion

In conclusion, health-care costs for treating hand and forearm injuries in young children have not been considered previously. When cases were sorted by mechanism and type of injury, fingertip injuries caused by jamming doors or other pinch objects were associated with the highest total health-care cost. Second highest total health-care cost, among the defined categories, were complex injuries caused by machines or rifles. Even though only 0.4–5% of the population, these cases induced a high cost for treatment of the individual child. Lost productivity was much less than observed in adults. In designing prevention strategies considering both the cost savings in the health care sector, and perhaps more importantly the avoided pain and suffering for the child, the results in this study indicate that two important groups are fingertip injuries (high total cost for the group) and complex injuries (high cost per case). Therefore, the costs for prevention should be considered in view of the question: "Cost per case or total cost?"

## Competing interests

The authors declare that they have no competing interests.

## Authors' contributions

All authors have contributed equally to the article.

## Pre-publication history

The pre-publication history for this paper can be accessed here:


